# Gender Disparity in Lipid Testing Among Over 0.5 Million Adults from Pakistan: Females are Tested Much Later Despite Higher LDL-Cholesterol Levels

**DOI:** 10.5334/gh.1401

**Published:** 2025-02-21

**Authors:** Amjad Nawaz, Madeeha Khan, Quratul Ain, Muhammad Amjad, Jaka Sikonja, Hijab Batool, Mohammad Iqbal Khan, Urh Groselj, Fouzia Sadiq

**Affiliations:** 1 Shifa Tameer-e-Millat University, H-8/4, Islamabad 44000, Pakistan; 2 Atta ur Rehman School of Applied Biosciences, National University of Sciences and Technology, H-12, Islamabad 44000, Pakistan; 3 Translational Genomics Laboratory, Department of Biosciences, COMSATS University Islamabad 44000, Pakistan; 4 Joint International Research Laboratory of Environment and Health, Ministry of Education, Guangdong Provincial Engineering Technology Research Center of Environmental Pollution and Health Risk Assessment, Department of Occupational and Environmental Health, School of Public Health, Sun Yat-sen University, Guangzhou 510080, China; 5 Department of Endocrinology, Diabetes, and Metabolic Diseases, University Children’s Hospital, University Medical Centre Ljubljana, Bohoriceva ulica 20, Ljubljana, Slovenia; 6 Faculty of Medicine, University of Ljubljana, Vrazov trg 2, Ljubljana, Slovenia; 7 Chemical Pathology, Chughtai Institute of Pathology, Lahore, Pakistan; 8 Department of Vascular Surgery, Shifa Tameer-e-Millat University, Shifa International Hospital Islamabad, H-8/4, Islamabad 44000, Pakistan

**Keywords:** Dyslipidemia, Gender Disparity, Cardiovascular disease, Pakistan, Opportunistic testing, Lipid testing

## Abstract

**Background and aims:**

Dyslipidemia is the major risk factor for atherosclerotic cardiovascular disease (ASCVD); therefore, its early diagnosis and treatment is necessary. While previous studies in Pakistan focused on general lipid profiles, investigations into gender disparities in lipid testing remain scarce. Therefore, the present study aims to explore the gender disparity in lipid testing and lipid levels among the adult Pakistani population.

**Methods:**

The lipid profile data was obtained from a tertiary care hospital and a diagnostic laboratory with centers across Pakistan. Dyslipidemia was defined based on the criteria provided by the National Cholesterol Education Program (NCEP) guidelines. Gender-based differences in lipid levels were analysed by copula decomposition, breaking down dyslipidemia differences into composition and structure effects.

**Results:**

A total of 577,489 adults were included in this study. The highest number of tests (n = 86,709, 14.6%) were conducted in individuals aged between 50 to 54 years. Greater number of males (n = 203,415, 64.3%) were tested before the age of 50 years compared to females (n = 113,030, 35.7%). Conversely, after the age of 50 years, number of tests increased notably among females (n = 137,541, 52.7%) compared to males (n = 123,503, 47.3%; p < 0.001). For all comparisons, significant differences were observed for low density lipoprotein cholesterol (LDL-C), triglycerides (TG), and high-density lipoprotein cholesterol (HDL-C) levels between males and females (p < 0.001), where average levels of LDL-C, TC and HDL-C were higher in females while average TG levels were higher in males.

**Conclusion:**

This study highlights the gender disparity in lipid testing in Pakistan, where females undergo lipid testing later in life, despite higher lipid levels compared to males.

## Introduction

Globally, more than 500 million individuals are affected by ASCVD, making it one of the leading causes of morbidity and mortality (1). A high proportion of the affected population, especially in lower- and middle-income countries, contributes significantly to the global burden of cardiovascular disease (CVD), where within Pakistan alone 15% of deaths in 2020 were caused by CVD (2,3).

Dyslipidemia, particularly elevated levels of low-density lipoprotein cholesterol (LDL-C), is the causal driver for the progression of atherosclerosis and the development of premature ASCVD (4). Late diagnosis/misdiagnosis and under-treatment along with physiological changes after menopause and pregnancy are great risk factors for ASCVD in women (5). Individuals with familial hypercholesterolemia (FH) are at higher risk of developing ASCVD due to lifelong elevations of LDL-C and generally remain underdiagnosed and undertreated (4,6,7) Women, particularly those with FH, are at a higher risk of developing ASCVD, mostly due to delayed diagnosis and undertreatment (8,9). Studies show that dyslipidemia is more common in women compared to men (10,11). Furthermore, women are less likely to seek healthcare due to sociocultural reasons mainly caregiving responsibilities, prioritizing the needs of others over their own health and stigma surrounding certain health issues (5,12). Consequently, dyslipidemia in women may remain undetected or untreated until reaching an advanced stage, increasing the risk of adverse cardiovascular outcomes. This underscores the critical importance of early detection and intervention, as treatment initiated in the early stages of dyslipidemia yields significantly better outcomes compared to therapies begun in later stages (13).

Previous studies conducted in Pakistan, focused primarily on the lipid profiles in the Pakistani population (14,15). In the context of Pakistan, research into lipid testing disparities between genders is indeed scarce. Therefore, the present study aims to examine gender differences in lipid testing within the adult Pakistani population and analyse the variations in lipid levels between genders.

## Patients and Methods

### Patients

**Figure 1:**
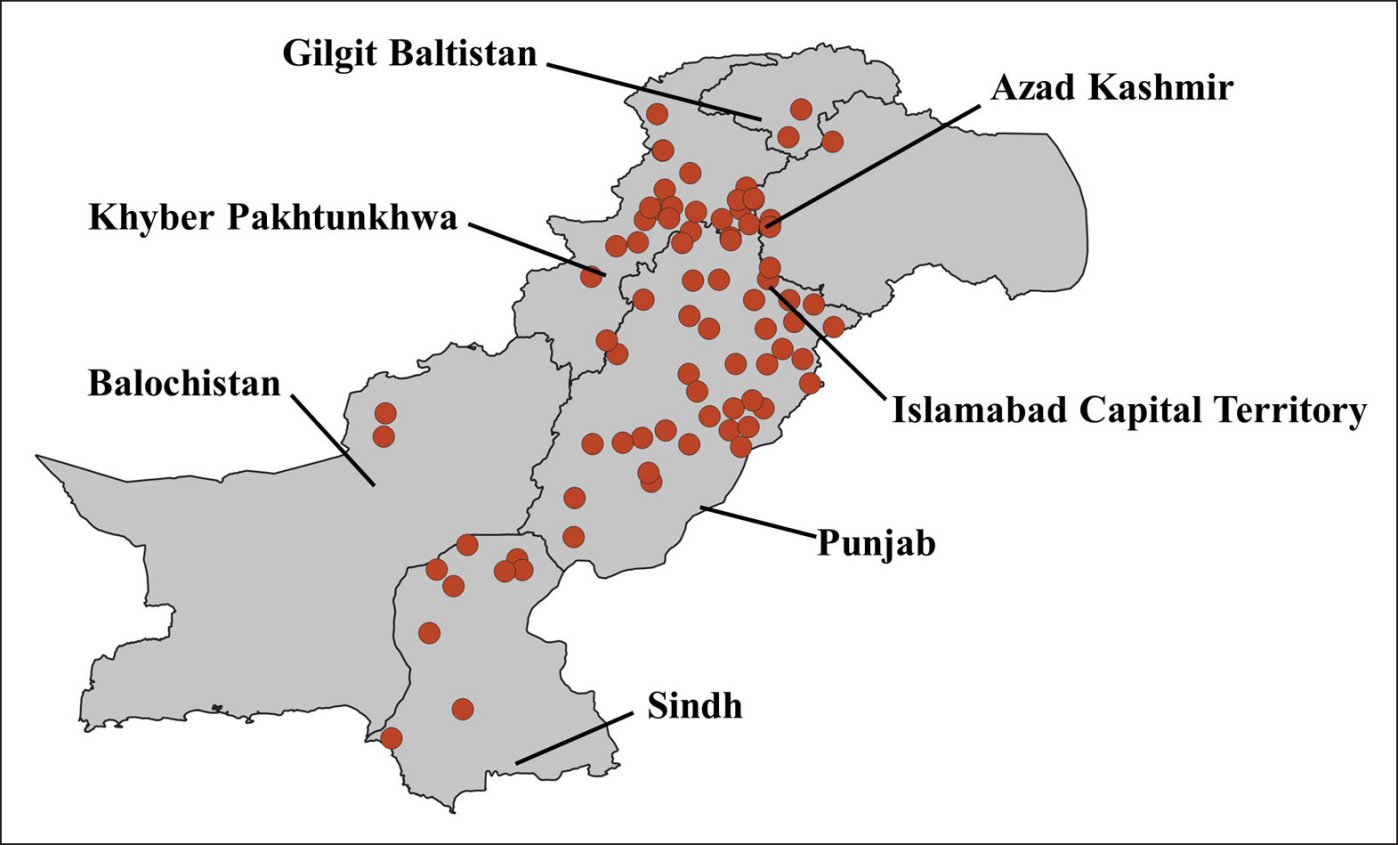
**Geographic distribution of lipid test locations across Pakistan.** This map illustrates the spatial distribution of lipid testing sites throughout Pakistan. Each red dot represents a location where lipid testing was taken, encompassing various cities and administrative divisions.

We performed a retrospective analysis of lipid testing data from laboratory databases of Shifa International Hospital, Islamabad and from a network of diagnostic centres and collection points of Chughtai Laboratories, Lahore. We retrieved anonymized data (n = 778,016), collected between March 2019 to March 2024, on lipid profile (Total cholesterol (TC), LDL-C, triglycerides (TG), high density lipoprotein cholesterol (HDL-C), age at measurement, gender (self-reported), city of residence, and year in which the test was conducted. In the case of repeated measurements of a single individual, only the value of the first test was included in the analysis. The data of individuals under 18 years (n = 9,934), multiple measurements of lipids (n = 159,841), missing city of residence (n = 1,879) and missing measurements of any lipid parameter (n = 28,873) were excluded from the study (Supplementary Figure 1). The visit location of individuals was extracted according to the subdivision (districts), and these subdivisions were traced back to their respective provinces and administrative units (Figure 1). The study was approved by Institutional Review Board and Ethics Committee (IRB&EC), Shifa Tameer-e-Millat University, Islamabad, Pakistan (IRB number 0323-22).

### Methods

#### Lipid profile testing

The lipid profile was analysed biochemically using homogenous enzymatic methods on separate analysers: the Cobas 8000 c502 module by Roche Diagnostics, USA, at Shifa International Hospital, and the Abbott Alinity ci analyser at Chughtai Laboratories. LDL-C levels were directly measured using a biochemical assay.

#### Dyslipidemia criteria

Dyslipidemia was defined by the National Cholesterol Education Program (NCEP) guidelines (LDL-C ≥130 mg/dL (3.36 mmol/L); TG ≥150 mg/dL (1.69 mmol/L); TC ≥200 mg/dL (5.17 mmol/L); or HDL-C < 40 mg/dL (1.03 mmol/L) (16). This criterion has been previously used for Pakistani population (14,15).

#### Decomposition and statistical analysis

The data was organized in Microsoft Excel and statistical analysis was performed using R version 4.2.3. Continuous variables were presented as mean, standard deviation (SD) and quantiles. The distribution of LDL-C, TG, HDL-C, and TC among genders was analysed using the Kolmogorov-Smirnov test. The null hypothesis represented the equality of the cumulative distribution function in females and males. Chi square test was performed to assess the association between genders and the age groups. The two-sided Mann-Whitney U test was used to analyse the difference between groups. The level of significance was set at 5% (p < 0.05) for all comparisons.

The copula decomposition, introduced by Rothe (2015) was employed to analyse the gender-based variations in lipid levels (17). The decomposition aimed to breakdown the gender differences in the measurements of elevated LDL-C, TG, TC, and low HDL-C. These analyses consist of two components: composition effect arising from variations in the relationship between explanatory variables, such as age, area of residence and year (in which the test was conducted) across genders, and a structure effect elucidating gender-related changes in the conditional distribution of the outcome variable (LDL-C, TG, TC, HDL-C) on the explanatory variables (age, area of residence, and year, in which the test was conducted). The significance of the results is determined by comparing estimates, greater estimates indicating stronger effect. The statistical significance of the estimates depends on the magnitude of the estimates for interpretation (18,19).

The details of the copula decomposition have been explained in the Supplementary materials section 1.1.

## Results

### General characteristics of the cohort

A total of 577,489 individuals were included in this study. Among these 242,530 (42.0%) were female and 334,959 (58.0%) were male. The mean ± SD age of the participants was 47.8 ± 13.7 years with male age (49.8 ± 13.9 years) and female age (47.1 ± 12.9 years). Most of the individuals belonged to the province of Punjab (n = 507,586, 87.9%) followed by Sindh (n = 25,513, 4.4%), Khyber Pakhtunkhwa (n = 16,215, 2.8%) and Islamabad Capital Territory (ICT, n = 13,097, 2.2%). The highest number of tests were conducted in districts Lahore (n = 250,438, 43.3%), followed by Gujranwala (n = 29,351, 5.0%), Faisalabad (n = 27,679, 4.7%), and Multan (n = 21,329, 3.6%) in Punjab; Islamabad (n = 13097, 2.2%) in ICT; Karachi (n = 18,564, 3.2%) in Sindh and Peshawar (n = 6,848, 1.1%) in Khyber Pakhtunkhwa province (Supplementary Table 1).

**Figure 2:**
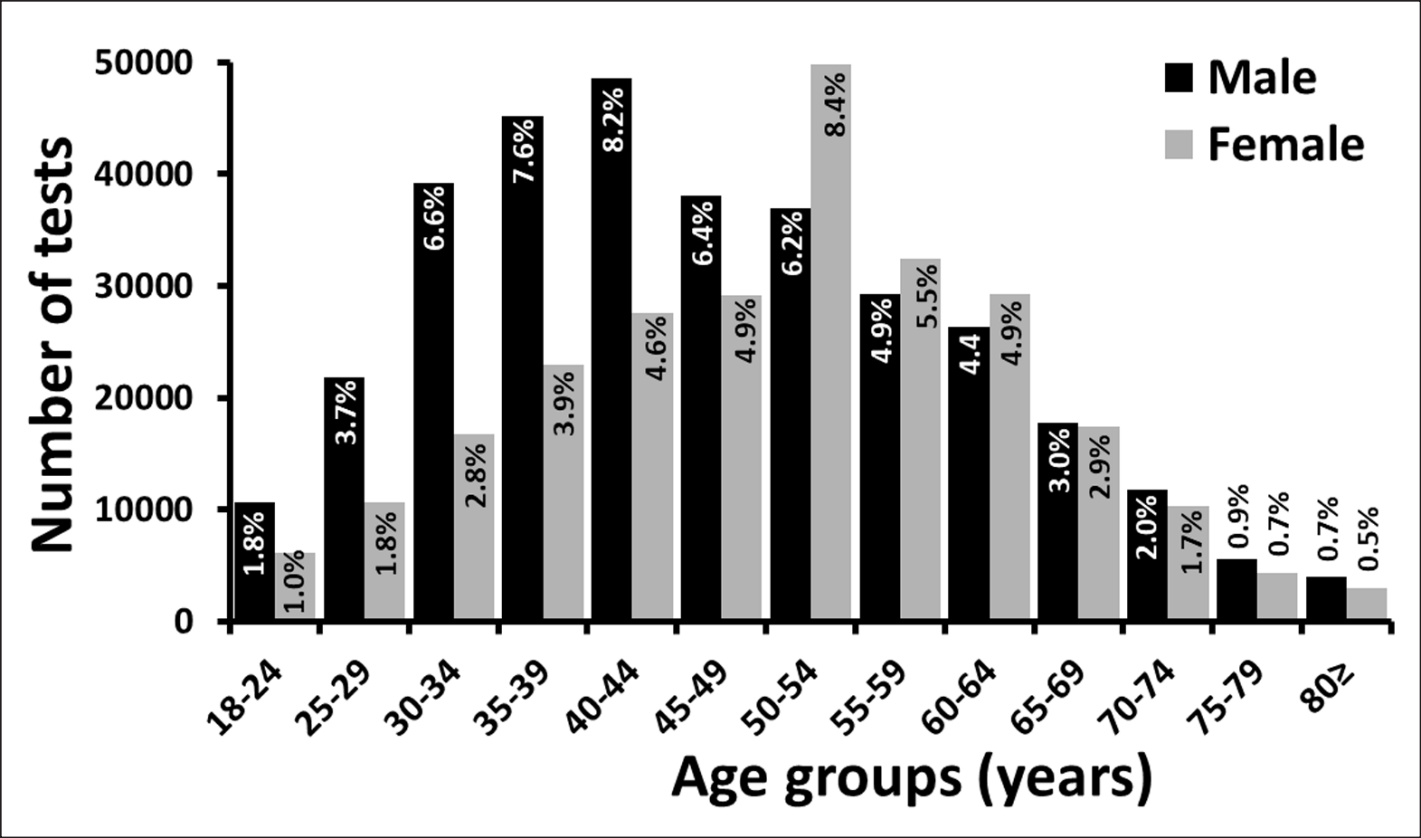
Gender and age specific results indicating the proportion of number of lipid profile tests across different age groups (n = 577,489).

The highest number of tests were conducted in the age range of 50 to 54 years (n = 86,709, 14.6%). A greater number of males (n = 203,415, 64.3%) were tested for lipid levels compared to females (n = 113,030, 35.7%) before the age of 50 years while the number of tests significantly increased in females (n = 137,541, 52.7%) after the age of 50 years compared to males (n = 123,503, 47.3%) (Figure 2). The differences in the number of tests were significant between gender and age (p value < 0.001).

For the overall population, the mean ± SD LDL-C levels for males were 125.5 ± 41.7 mg/dL and 123.1 ± 42.4 mg/dL for females (p < 0.001). The mean ± SD TG, TC and HDL-C levels varied significantly among genders (p < 0.001) (Table 1).

**Table 1:** Characteristics of the dyslipidemic population according to gender (n = 577,489).


	N (%)	MEAN ± SD	CONFIDENCE INTERVAL (CI 95%)	PROPORTION OF TESTED INDIVIDUALS	CASES PER 10,000

**LDL-C ≥130 mg/dL (3.36 mmol/L)**					

Overall	251,599 (43.6)	161.2 ± 30.3	161.1–161.3	1:2.2	4,400

Male	143,175 (56.9)	160.9 ± 30.8	160.7–161.0		

Female	108,424 (43.1)	161.7 ± 29.6	161.5–161.8		

**TG ≥150 mg/dL (1.69 mmol/L)**					

Overall	305,412 (52.8)	262.1 ± 215.6	261.4–262.9	1:1.8	5,300

Male	187,857 (61.5)	268.1 ± 219.6	267.1–269.1		

Female	117,555 (38.5)	252.6 ± 208.6	251.4–253.8		

**TC ≥200 mg/dL (5.17 mmol/L)**					

Overall	206,105 (35.6)	233.2 ± 36.2	233.0–233.3	1:2.8	3,600

Male	116,054 (56.3)	233.1 ± 37.3	232.9–233.3		

Female	90,051 (43.7)	233.2 ± 34.6	232.9–233.4		

**HDL-C < 40 mg/dL (1.03 mmol/L)**					

Overall	307,834 (53.3)	32.8 ± 4.8	32.7–32.8	1:1.8	5,300

Male	213,521 (69.4)	32.7 ± 4.6	32.7–32.8		

Female	94,313 (30.6)	33.7 ± 5.1	32.9–33.0		


### Overall dyslipidemia burden

Based on the NCEP criteria for dyslipidemia, 43.6% individuals had high LDL-C, 52.8% had high TG, 35.6% had high TC and 53.3% had low HDL-C (Table 1). The mean ± SD for high LDL-C was 161.2 ± 30.3 mg/dL while for high TG, TC and low HDL-C, it was 262.1 ± 215.6 mg/dL, 233.2 ± 36.2 mg/dL, and 32.8 ± 4.8 mg/dL respectively (Table 1).

### Proportion of dyslipidemia

**Figure 3:**
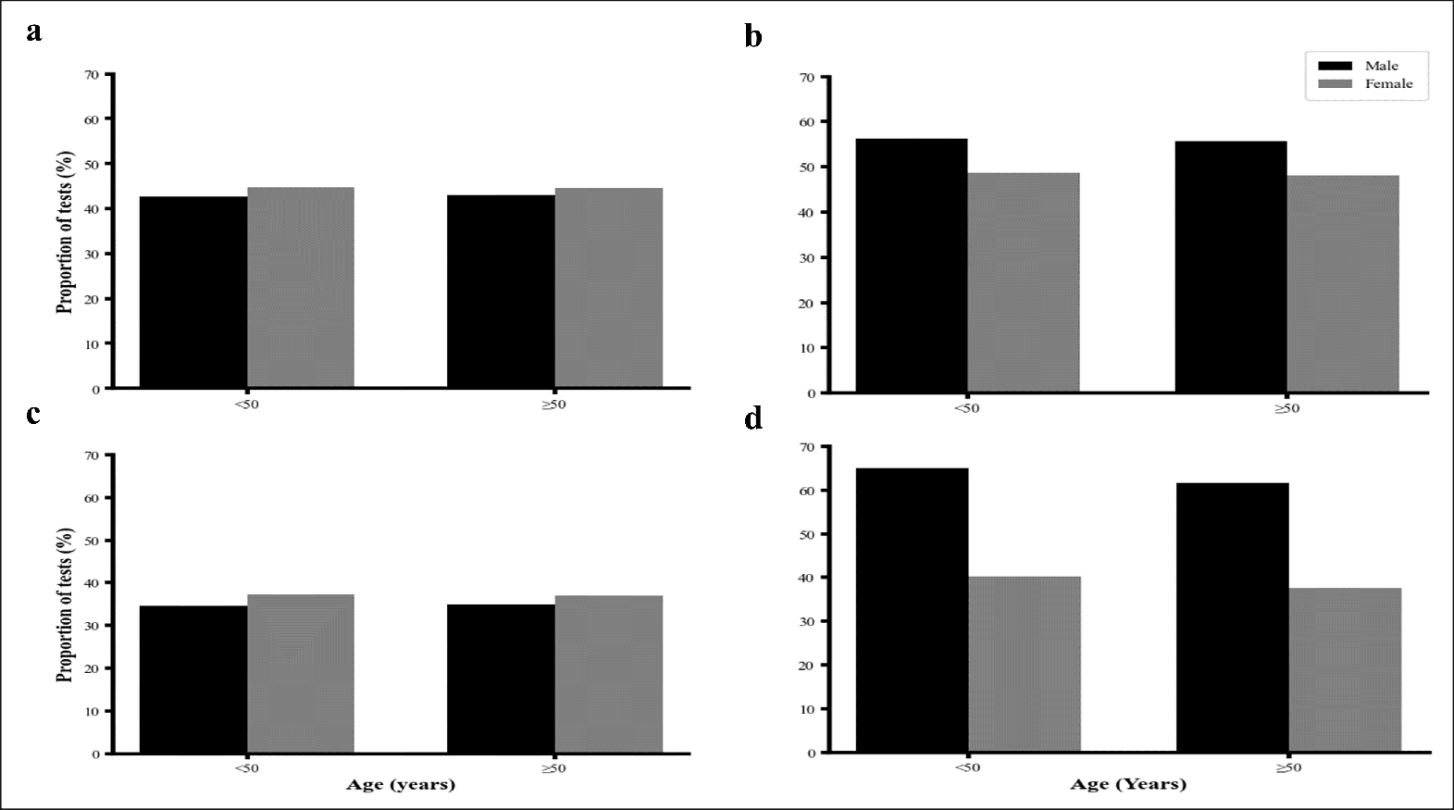
**Proportion of dyslipidemia** a) Low density lipoprotein cholesterol (LDL-C), b) Triglycerides (TG), c) Total cholesterol (TC), d) High density lipoprotein cholesterol (HDL-C) classified based on gender and age categories (<50 years and ≥50 years).

Among all individuals that underwent lipid testing, females exhibited a higher proportion of elevated LDL-C (44.8% vs. 42.6%, p-value < 0.001) and TC (37.2% vs. 34.5%, p-value < 0.152) compared to males before the age of 50 years. Conversely, males had a higher proportion of elevated TG (56.3% vs. 48.8%, p-value < 0.001) and low HDL-C (65.1% vs. 40.3%, p-value < 0.001) compared to females in the same age group. After the age of 50 years, similar trends were observed where females had higher proportion for elevated LDL-C (44.6% vs. 43.0%, p-value < 0.001), TC (37.1% vs. 34.9%, p-value < 0.001), and males continuing to have a higher proportion of elevated TG (55.7% vs. 48.2%, p-value < 0.001) and HDL-C (61.6% vs. 37.7%, p-value < 0.001) compared to females (Figure 3).

The mean LDL-C (161.7 ± 29.6 mg/dL), TC (233.1 ± 37.3 mg/dL) and HDL-C (37.7 ± 5.1 mg/dL) levels were higher in females compared to males (LDL-C (160.9 ± 30.8 mg/dL), (TC (233.1 ± 37.3 mg/dL), (HDL-C (32.7 ± 4.6 mg/dL). The mean TG levels were higher in males (268.1 ± 219.6 mg/dL) compared to females (252.6 ± 208.6 mg/dL). The gender differences were statistically significant for age, TC, LDL-C, TG and HDL-C (p < 0.001) (Table 1).

Among individuals with dyslipidemia, majority of individuals belonged to Lahore (LDL-C (n = 112,571, 44.7%), TG (n = 132,453, 43.3%), TC (n = 90,260, 43.8%), and HDL-C (n = 131,672, 42.7%) followed by Gujranwala (LDL-C (n = 13,216, 5.2%), TG (n = 15,416, 5.0%), TC (n = 10,500, 5.0%), and HDL-C (n = 15,340, 4.9%) and Faisalabad (LDL-C (n = 12,569, 5.0%), TG (n = 14,008, 4.5%), TC (n = 10,165, 4.9%), and HDL-C (n = 12,545, 4.0%). The burden of dyslipidemia deducted according to cities of Pakistan is presented in Supplementary Figure 2).

### Distribution and decomposition of lipid parameters by gender

**Figure 4:**
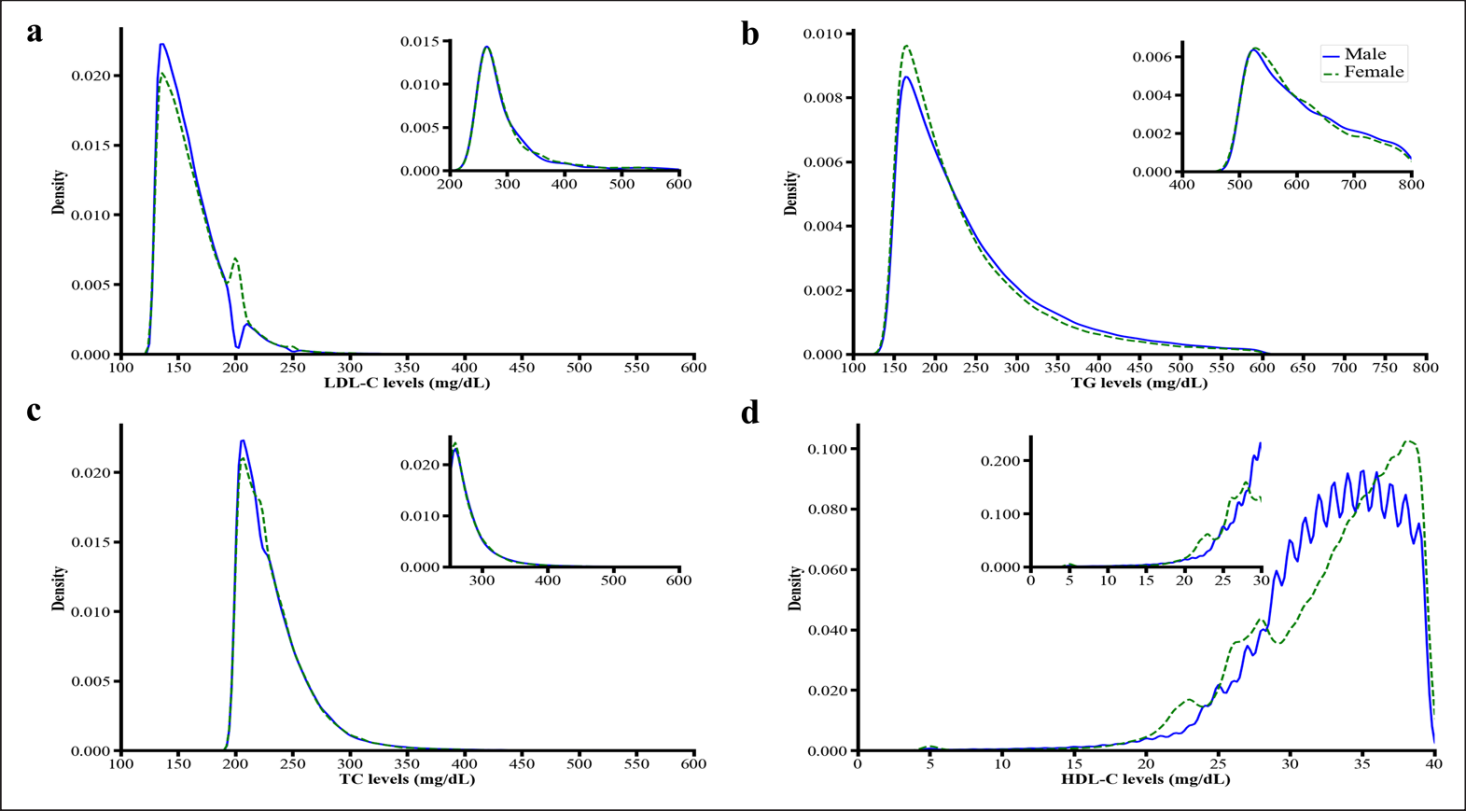
Kernel density estimates of **a)** Low density lipoprotein cholesterol (LDL-C), **b)** Triglycerides (TG), **c)** Total cholesterol (TC), **d)** High density lipoprotein cholesterol (HDL-C). Lipid levels on x-axis, density estimates on y-axis. The insets represent kernel density estimates at extreme levels of lipids. Solid line indicates male and dotted line indicates female. Higher peaks reflect greater data points while lower densities reflect fewer data points in that area.

The kernel density estimates (KDE) were used for the visualization of the distribution of continuous variables (LDL-C, TG, TC, and HDL-C) and for comparing these distributions between males and females (Figure 4). The smaller differences were observed in the lipid levels between the genders near the central portion of the curves and towards the tails of the curves. The results of the Kolmogorov-Smirnov test showed that the distribution of LDL-C, TG, TC, and HDL-C were not normally distributed between genders.

The estimated results of the decomposition of TC, LDL-C, TG and HDL-C on average and at quantiles are presented in Supplementary Table 2 (a, b, c, d). The negative sign indicates higher mean levels in the females while the positive sign indicates higher mean levels in males.

LDL-C: The average difference in elevated LDL-C levels between the two groups was –0.927 mg/dL, with females showing higher mean levels than males at the average, 50th, and 90th quantiles. This difference was decomposed into structure effect (–0.847 mg/dL) and composition effect (–0.081 mg/dL). The age, and area of residence impacted these differences.

TG: The average difference in elevated TG levels between the two groups was 16.096 mg/dL. The structure effect decreased with each quantile, while the composition effect increased on average as well as quantiles. Age and area of residence had greater impact on TG levels based on the composition effect.

TC: The mean of elevated levels of TC increased in females (–1.350 mg/dL) on average as well as within quantiles. The structure and composition effect were negative and significantly decreased on average with all quantiles.

HDL: The mean elevated levels of TC increased by (–0.229 mg/dL) in females on average, as well as across all quantiles. The composition effects (0.624 mg/dL) were increased on average and all quantiles.

The marginal effect was further decomposed into direct effect of each explanatory variable which demonstrated that age and area of residence were significantly associated with high levels of TC, LDL-C, and TG. The two-way interaction effects were not significant, and the interaction effects were zero indicating that the explanatory variables have no impact on the response variables. Therefore, interaction effects were not considered.

## Discussion

This study was conducted to evaluate the gender disparity in lipid testing among the Pakistani adult population visiting hospitals or diagnostics labs. It is worth noting that healthcare disparities based on gender are more apparent in women than in men. It is essential for healthcare providers, particularly those specializing in cardio-obstetrics, to better understand how women access cares to enhance diagnostic rates (20).

Most of our study participants who underwent lipid testing were males (58.0%) as compared to females (42.0%). A recent study conducted in Pakistan by Farhad *et al*. also reported that 58.8% of individuals visiting diagnostic labs for lipid testing were men, which aligns with our findings regarding gender disparities in lipid screening practices in Pakistan (21). Similarly, another study conducted in Pakistan also reported late diagnosis among females compared to males (22). Our data further reveals a notable age-related pattern in lipid testing between genders. Men tended to undergo lipid tests at younger ages, while women were more likely to seek lipid testing after the age of 50 years. This trend suggests reactive testing (lipid testing) in women when they get older and have already experienced a CVD event. Unfortunately, from the available data, where we do not have information on clinical characteristics and CVD events, it is difficult to claim this, but we can assume that it is true. At the same time, we must keep in mind that we have used data on first tests (i.e., the first lipid measurements), which clearly indicates that women are at a disadvantage when it comes to the early detection of dyslipidemia, one of the main causes of CVD. Due to the later first testing for dyslipidemia, women are therefore deprived of adequate primary prevention and CVD event prevention. This gender-based discrepancy in lipid testing patterns may have significant implications for early detection and management of dyslipidemia. Kolovou and colleagues (2009) emphasized that gender-specific variations in lipid profiles are crucial for accurately diagnosing and preventing atherosclerosis (23). The observed differences in testing patterns could potentially lead to delayed diagnoses in women, highlighting the need for targeted awareness campaigns and screening initiatives.

Significant disparities were observed in the average TC, LDL-C, TG and HDL-C levels among males and females. Age and area of residence were identified as the main driver of the composition effect based on the copula decomposition. The differences observed on the basis of area of residence are particularly important for the Pakistani population because households in rural areas often experience deprivation in health, education, financial status, and food security (24,25). The number of lipid tests in females increased after 50 years, which is consistent with the results of Gupta and colleagues (2016) in the Indian population and other studies from world (26,27). Our findings of gender and age-related associations with dyslipidemia are similar to those reported in the Chinese population (28).

Our findings revealed a concerning prevalence of dyslipidemia burden in the studied Pakistani population, with 43.6% of individuals exhibiting high LDL-C, 52.8% high TG, 35.6% high TC, and 53.3% low HDL-C levels, these results indicate a significant burden of lipid abnormalities. Another study conducted in Pakistan showed the burden of high TC in 39.0%, high TG in 49.0% and high LDL-C in 38.0% of the population (14). Comparing our findings to other Asian populations, we observe notable differences. Joshi and colleagues (2014) reported lower prevalence rates in India, with 14.0% of individuals having high TC, 11.8% high LDL-C, 29.5% high TG, and 72.3% low HDL-C (29). Similarly, Zhang and colleagues (2018) found lower rates in the Chinese population, with high TC at 6.9%, high LDL-C at 8.1%, high triglycerides at 13.8%, and low HDL-C at 20.4% (30). The markedly higher prevalence of dyslipidemia in our Pakistani cohort, particularly in LDL-C, TG, and TC levels, highlights a pressing public health concern. This disparity may be attributed to various factors, including genetic predisposition, dietary habits, lifestyle choices, and potentially inadequate awareness or management of cardiovascular risk factors in the Pakistani population.

Sociocultural factors could be one of the major reasons for this difference where women are less likely to seek healthcare (5). Women in Pakistan face several challenges when it comes to accessing healthcare, such as significant household duties, limited decision-making power, difficulties in traveling, and tendency to prioritize the health concern of male family members (12,31). Late lipid profile testing and inadequate treatment, especially for women, increase the risk of cardiovascular disease, therefore it is important to assess the cardiovascular risk early to begin prompt therapy in women.

There are some limitations of the present study. The data utilized for this study was solely obtained from databases of diagnostic networks so data of parameters such as body mass index, co-morbidities, and family medical history was not available. Furthermore, it is also not known whether lipid tests were ordered for screening or management purposes, which could have introduced a selection bias, due to which the sample included in this study may not reflect the characteristics of the general Pakistani population limiting the generalizability of our findings. Additionally, we obtained our data from the largest private diagnostic setup, which may have excluded low-income individuals who typically seek care in government hospitals. These factors could potentially impact the generalizability of our results. Moreover, it should be noted that we did not have access to data on lipid-lowering therapy, thus limiting our ability to analyse therapy-related outcomes in this context.

## Conclusion

In conclusion, our study reveals that despite being at elevated risk, females are tested late leading to missed treatments. These findings highlight the importance of implementing comprehensive screening programs and developing gender-specific strategies for the screening and management of dyslipidemia in the country, which could be instrumental in reshaping public health policies leading to gender equity in healthcare.

## Additional File

The additional file for this article can be found as follows:

10.5334/gh.1401.s1 Supplementary Material. This includes detailed methodology for decomposition analysis, along with additional tables and figures supporting the main findings.
