# Supplementary material for: Gender Disparity in Lipid Testing Among Over 0.5 Million Adults from Pakistan: Females are Tested Much Later Despite Higher LDL-Cholesterol Levels
Source: Glob Heart. 2025 Feb 21;20(1):16. doi: 10.5334/gh.1401 (PMC11844004; doi:10.5334/gh.1401)
Supplement: Supplementary Material. — This includes detailed methodology for decomposition analysis, along with additional tables and figures supporting the main findings. [file gh-20-1-1401-s1.pdf]

Supplementary Material

Gender Disparity in Lipid Testing Among Over 0.5 Million Adults from Pakistan: Females Are Tested Much Later Despite Higher LDL-Cholesterol Levels

Amjad Nawaz, MPhil[1], Madeeha Khan, MS[1,2], Quratul Ain, MPhil[1,3], Muhammad Amjad, PhD[4], Jaka Sikonja, MD[5,6], Hijab Batool, FCPS[7], Mohammad Iqbal Khan, FRCS[9], Urh Groselj, MD, PhD[5,6**], Fouzia Sadiq, PhD[1*]

[1]Directorate of Research, Shifa Tameer-e-Millat University, Islamabad, Pakistan
[2]Atta ur Rehman School of Applied Biosciences, National University of Sciences and Technology, Islamabad
[3]Translational Genomics Laboratory, Department of Biosciences, COMSATS University Islamabad,
Pakistan
[4]Joint International Research Laboratory of Environment and Health, Ministry of Education, Guangdong Provincial Engineering Technology Research Center of Environmental Pollution and Health Risk Assessment, Department of Occupational and Environmental Health, School of Public Health, Sun Yat-sen University, Guangzhou 510080, China
[5]Department of Endocrinology, Diabetes, and Metabolic Diseases, University Children's Hospital,
University Medical Centre Ljubljana, Bohoriceva ulica 20, Ljubljana, Slovenia
[6]Faculty of Medicine, University of Ljubljana, Vrazov trg 2, Ljubljana, Slovenia
[7]Chemical Pathology, Chughtai Institute of Pathology, Lahore, Pakistan
[8]Department of Vascular Surgery, Shifa Tameer-e-Millat University, Shifa International Hospital Islamabad, Pakistan

1. Decomposition Methodology

The copula decomposition, introduced by Rothe, 2015 was utilized to analyse the gender-based variations in lipid levels. The decomposition aimed to breakdown the gender differences in the measurements of elevated TC, LDL-C, TG, and low HDL-C. These analyses consist of two components: composition effect arising from variations in the relationship between explanatory variables such as age, area of residence and time period across genders, and a structural effect elucidating gender-related changes in the conditional distribution of the outcome variable (TC, LDL-C, TG, HDL-C) given the explanatory variables (age, province, and time period). The detailed methodology added in the supplementary material that provides a comprehensive overview of decomposition analysis.

The implementation of the decomposition method involves the calculation of various functions and parameters, including univariate cumulative distribution functions (CDFs), the conditional CDF of Y^g |X^g (Yg represents the response variable and Xg, explanatory variable) and the parameters of copula functions. The decomposition method estimates the different functions and parameters and to decompose the differences between the groups (female and male).

$$\Omega_y^v = v(F_y^f) - v(F_y^m) \qquad (1)$$

Where, $v(F_y^f)$ and $v(F_y^m)$ estimate the characteristic of the distribution at mean, median and different quantiles for female and male respectively. These function and parameters are univariate cumulative distribution function, CDF of (Y^g/X^g). The conditional cumulative distribution of Y^g/X^g is called multivariate function, it depends on the number of explanatory variables. The estimated CDF is based on the distribution regression (Foresi & Peracchi, 1995; Rothe, 2015). This regression model assumes that:

$$F_{Y|X}^t(y,x) = \Phi\left(x'\delta^t(y)\right) \quad (2)$$

where Φ(.) represents standard normal CDF and δ^t (y) denotes finite-dimensional parameter estimated by Maximum Likelihood Estimator (MLE).

The final step in implementing the decomposition procedure involves utilizing a copula function (Rothe, 2015). This copula belongs to a parametric class (gaussian distribution) determined by a k-dimensional parameter θ. To estimate the copula's characteristic parameters, the minimum distance estimator, as defined by Weiß (2011) was employed.

$$\widehat{\theta_t} = arg\ \theta min \sum_{i=1}^{n_t}\left(\hat{F}X1t(X_{1i}^t, \ldots\ldots, X_{di}^t\right) - C_\theta\left(\hat{F}X1t(X_{1i}^t), \ldots\ldots, \hat{F}Xdt(X_{di}^t))\right) \quad (3)$$

$$C_\Sigma(u) = \Phi_\Sigma^d\left(\Phi^{-1}(u_d)\right) \quad (4)$$

Various parametric copula functions serve different purposes (Trivedi and Zimmer, 2007). Yet, when choosing a suitable function, it's essential to ensure sufficient flexibility to encompass all potential forms of dependence. Additionally, we must consider that our variables comprise a mix of continuous and discrete forms. In addressing these considerations, we opt for the Gaussian copula model.

Where $\Phi_\Sigma^d(.)$ represents the CDF of a d-variate standard normal distribution with correlation matrix Σ, and $\Phi^{-1}(.)$ known as an inverse function of the standard normal distribution function Φ(.) The parameters $\theta = \Sigma$ determine the pattern of dependence among the explanatory variables (age, province, time period).

1.1. References

1. Foresi, S., & Peracchi, F. (1995). The conditional distribution of excess returns: An empirical analysis. *Journal of the American Statistical Association*, 90(430), 451–466.
2. Rothe, C. (2015). Decomposing the Composition Effect: The Role of Covariates in Determining Between-Group Differences in Economic Outcomes. *Journal of Business & Economic Statistics*, 33(3), 323–337.
3. Weiß, G. (2011). Copula parameter estimation by maximum-likelihood and minimum-distance estimators: a simulation study. *Computational Statistics*, *26*(1), 31–54.

2. Supplementary tables

Supplementary Table 1: Number and percentage of top 20 districts for lipid profile tests performed (n=577,489)

Sr No.	City	Total number of tests	Percentage (%)
1	Abbotabad	3,361	0.6
2	Bahawalpur	5,797	1.0
3	Faisalabad	27,679	4.7

4	Gujranwala	29,351	5.0
5	Gujrat	6,001	1.0
6	Islamabad	13,097	2.2
7	Jhang	3,737	0.6
8	Karachi	18,564	3.2
9	Kasur	5,574	0.9
10	Lahore	250,438	43.3
11	Multan	21,329	3.6
12	Okara	3,070	0.5
13	Peshawar	6,848	1.1
14	Rahim yar Khan	4,505	0.7
15	Rawalpindi	6,136	1.1
16	Rawalpindi	5,242	0.9
17	Sahiwal	5,715	1.0
18	Sargodha	7,319	1.3
19	Sheikhupura	8,730	1.5
20	Sialkot	7,406	1.3

Supplementary Table 2: Estimated decomposition results

(a) Estimated decomposition results of total cholesterol (TC)

	**Mean**		**10th Quantile**		**50th Quantile**		**90th Quantile**	
	**Estimated value**	**Standard error**	**Estimated value**	**Standard error**	**Estimated value**	**Standard error**	**Estimated value**	**Standard error**
Total difference	-1·350	0·387	-0·361	0·213	-2·347	0·109	-3·734	1·549
Structure effect	-0·240	0·407	-0·141	0·222	-1·626	0·095	-3·347	1·596
Composition effect	-0·110	0·020	-0·220	0·009	-0·721	0·014	-0·297	0·048
**Composition effect:**								
Dependence effect	-0·004	0·004	0·008	0·007	0·003	0·016	0·003	0·037
Marginal effect	-0·106	0·015	-0·228	0·001	-0·724	0·002	-0·301	0·011
**Direct contribution to composition effect**								
Age	-0·070	0·005	0·004	0·003	0·007	0·007	-0·009	0·008
Area of residence	-0·025	0·011	-0·028	0·002	-0·222	0·004	-0·218	0·019

Year	0·011	0·000	-0·203	0·000	-0·525	0·001	-0·089	0·000

(b): Estimated decomposition results of low-density lipoprotein cholesterol (LDL-C)

	Mean		10th Quantile		50th Quantile		90th Quantile	
	**Estimated value**	**Standard error**	**Estimated value**	**Standard error**	**Estimated value**	**Standard error**	**Estimated value**	**Standard error**
Total difference	-0·927	0·504	-0·123	0·087	-0·767	0·047	-1·861	0·582
Structure effect	-0·847	0·450	-0·118	0·087	-0·738	0·113	-1·665	0·476
Composition effect	-0·081	0·054	-0·006	0·000	-0·028	0·067	-0·196	0·105
**Composition effect:**								
Dependence effect	0·002	0·007	0·001	0·000	0·004	0·001	-0·002	0·001
Marginal effect	-0·083	0·047	-0·007	0·002	-0·033	0·060	-0·199	0·105
**Direct contribution to composition effect**								
Age	-0·044	0·009	-0·007	0·002	-0·032	0·004	-0·161	0·037

Area of residence	-0·035	0·039	-0·016	0·001	-0·062	0·056	-0·034	0·069
Year	-0·004	0·000	-0·002	0·000	-0·003	0·001	-0·004	0·001

(c): Estimated decomposition results of triglycerides (TG)

	**Mean**		**10th Quantile**		**50th Quantile**		**90th Quantile**	
	**Estimated value**	**Standard error**	**Estimated value**	**Standard error**	**Estimated value**	**Standard error**	**Estimated value**	**Standard error**
Total difference	16·096	1·960	1,331	0·789	8·604	2·275	29·098	7·106
Structure effect	-46·571	1·903	-3·878	0·791	-17·537	2·164	-96·437	6·723
Composition effect	62·667	0·057	5·209	0·001	26·141	0·111	125·534	0·382
**Composition effect:**								

Dependence effect	-0·071	0·005	-0·003	0·001	-0·014	0·000	-0·086	0·009
Marginal effect	62·738	0·051	5·212	0·000	26·155	0·111	125·620	0·391
**Direct contribution to composition effect**								
Age	63·709	0·099	4·780	0·017	27·785	0·001	-123·845	0·077
Area of residence	-0·547	0·150	0·412	0·017	0·131	0·112	0·197	0·467
Year	0·250	0·000	-0·017	0·000	0·035	0·001	1·050	0·002

(d): Estimated decomposition results of high-density lipoprotein cholesterol (HDL-C)

	**Mean**		**10th Quantile**		**50th Quantile**		**90th Quantile**	
	**Estimated value**	**Standard error**	**Estimated value**	**Standard error**	**Estimated value**	**Standard error**	**Estimated value**	**Standard error**
Total difference	-0·229	0·066	1·033	0·133	-0·830	0·101	-0·430	0·022

Structure effect	-0·853	0·063	-0·242	0·130	-0·415	0·101	-0·494	0·021
Composition effect	0·624	0·005	1·275	0·005	0·415	0·006	0·064	0·001
**Composition effect:**								
Dependence effect	0·006	0·001	0·015	0·002	0·002	0·001	0·000	0·000
Marginal effect	0·621	0·005	1·260	0·003	0·413	0·005	0·064	0·001
**Direct contribution to composition effect**								
Age	0·021	0·005	-0·003	0·004	0·017	0·006	0·007	0·001
Area of residence	0·602	0·000	1·257	0·001	0·398	0·001	0·058	0·001
Year	-0·002	0·000	-0·004	0·000	0·000	0·000	0·000	0·000

3. **Supplementary figures**

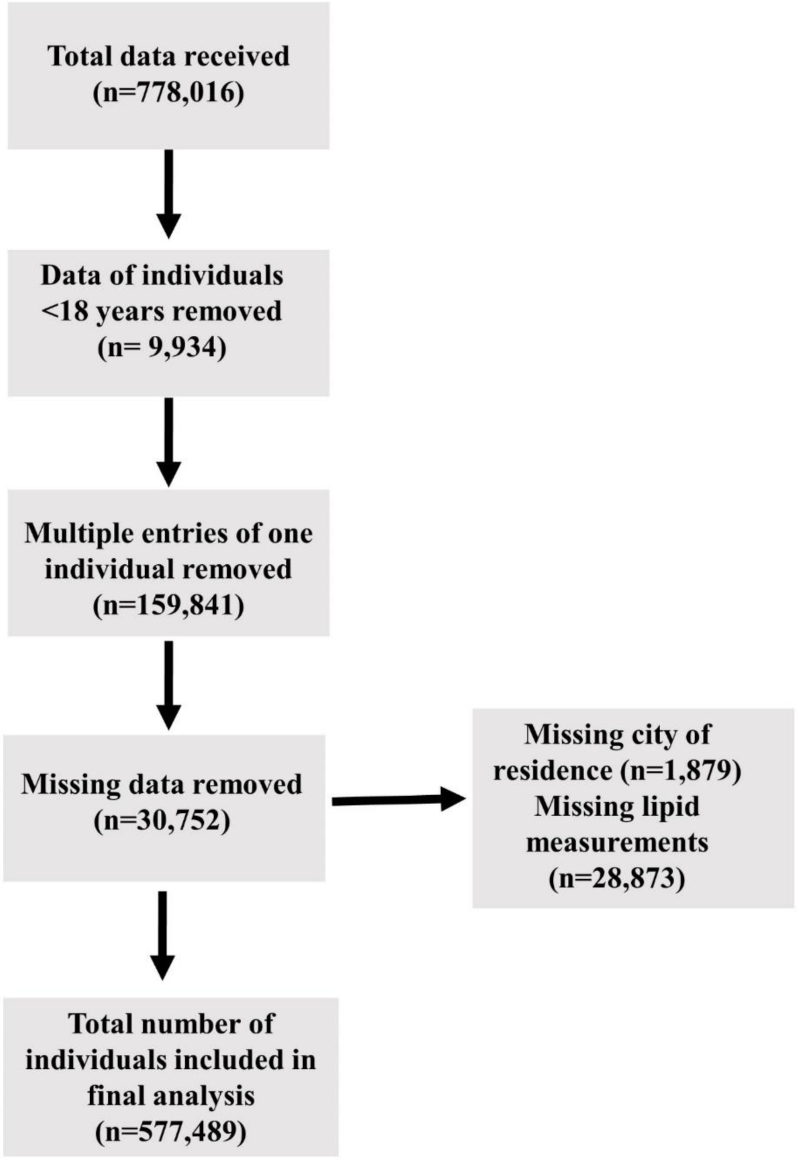

Supplementary figure 1: Flow diagram for the number of individuals included in the final analysis

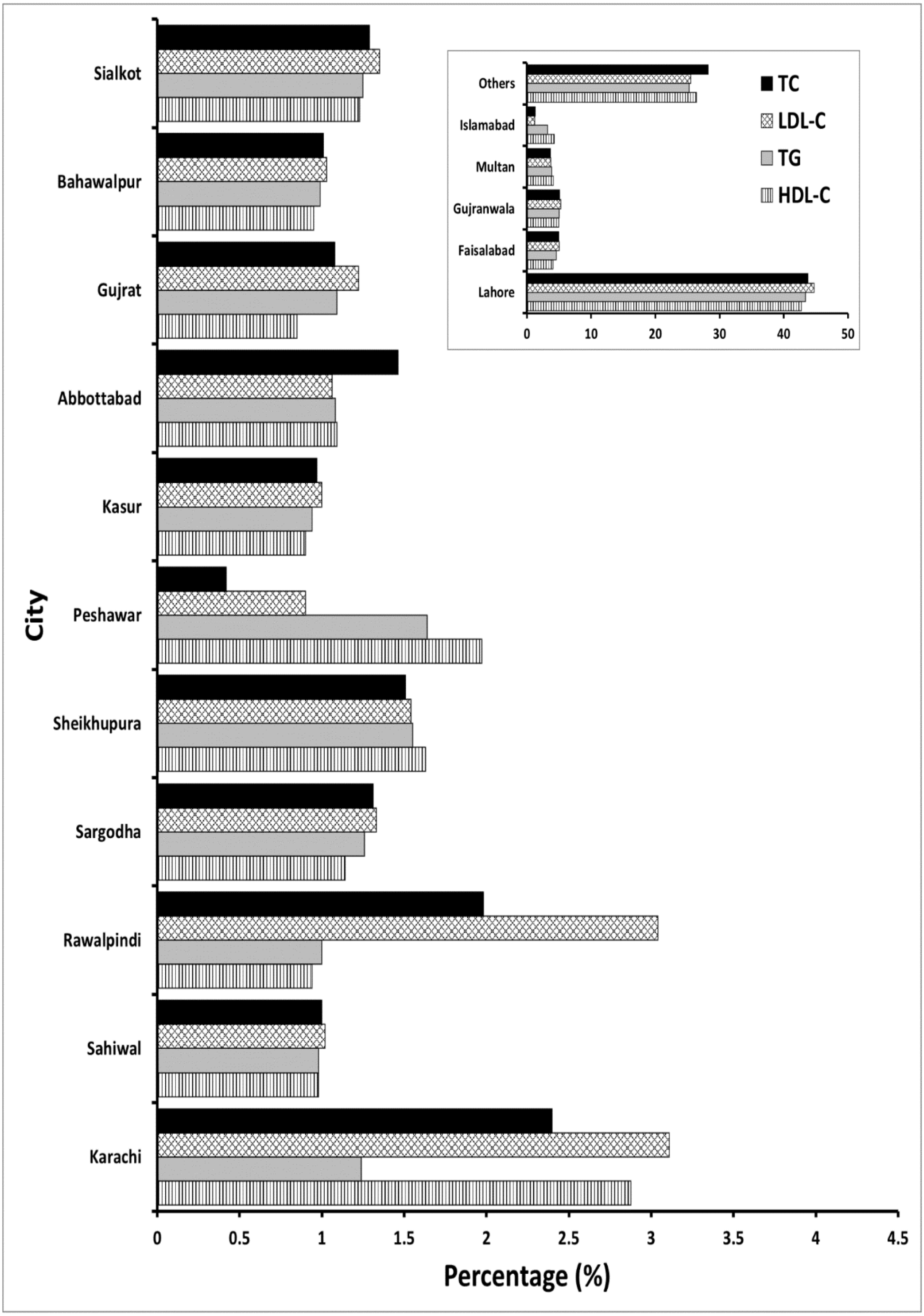

Supplementary Figure 2: Rate of dyslipidemia according to top 16 cities of Pakistan
